# Habitat Complexity Reduces the Feeding Strength of Freshwater Predators

**DOI:** 10.1002/ece3.72258

**Published:** 2025-10-21

**Authors:** Mireia Aranbarri, Lorea Flores, Ioar de Guzmán, Aitor Larrañaga, Björn C. Rall, Julia Reiss

**Affiliations:** ^1^ Laboratory of Stream Ecology, Department of Plant Biology and Ecology, Faculty of Science and Technology University of the Basque Country, UPV/EHU Bilbao Spain; ^2^ INRAE, UMR 1224, Ecologie Comportementale et Biologie des Populations de Poissons, Aquapôle, Quartier Ibarron Saint‐Pée sur Nivelle France; ^3^ Aquatic Ecology and Evolution, Department of Biology University of Konstanz Konstanz Germany; ^4^ Centre for Pollution Research and Policy Brunel University of London Uxbridge UK

**Keywords:** ecosystem stability, functional response, habitat structure, predation, prey, trophic interactions

## Abstract

The physical structure of an environment potentially influences feeding interactions among organisms, for instance, by providing refuge for prey. We examined how habitat complexity affects the functional feeding response of an ambush predator (damselfly larvae *Ischnura elegans*) and a pursuit predator (backswimmer *Notonecta glauca* ) feeding on the isopod *Asellus aquaticus* . We ran experiments in aquatic microcosms with an increasing number of structural elements (0, 2, or 3 rings of plastic plants in different spatial configurations), resulting in five habitat complexity levels. Across these levels, predators were presented with different prey densities to determine the functional response pattern. The experimental design and analysis allowed us to test for effects of structure presence, amount, and complexity level on functional response in one pass, without confounding predictors. Across all complexity levels, the feeding for both predators was best described by a type II functional response model, and habitat drove feeding strength. Regarding the latter, the predators showed different responses to the complexity treatments. The overall feeding rate of *I. elegans* was mainly explained by the absence versus presence of structure. Yet, in the case of *N. glauca* , feeding rate was strongly dependent on habitat complexity with the predator showing a unique maximum feeding rate (i.e., the inverse of the handling time) for each complexity level and a decreasing attack rate with increasing amount of habitat. On average, prey consumption by both predators was reduced when complex structures were present, compared to the ‘no habitat structure’ environment (e.g., consumption more than halved for some treatments). Our findings demonstrate that habitat complexity dampens feeding rates and therefore plays a key role in the stability of freshwater ecosystems.

## Graphical abstract

We investigated how habitat complexity influences the feeding responses of an ambush predator (*Ischnura elegans*) and a pursuit predator ( *Notonecta glauca* ) preying on *Asellus aquaticus* . Feeding rates of *I. elegans* were primarily affected by the presence or absence of structure, whereas *N. glauca* exhibited a unique maximum feeding rate and a declining attack rate with increasing habitat complexity. Overall, both predators consumed less prey in complex habitats, with consumption reduced by more than half in some treatments.

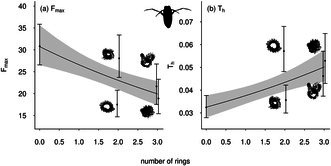

## 1 Introduction

The physical structure of an environment is a crucial factor in shaping community composition and the ecological processes driven by living organisms. Habitat complexity has been shown to influence population density, body size, and species richness of invertebrates in both terrestrial and aquatic ecosystems (Flores et al. 2016; Soukup et al. 2022; White and Walsh 2020). Furthermore, habitat complexity influences the interactions among organisms by providing refuge, thereby reducing predation risk for prey, especially when prey densities are low (Barrios‐O'Neill et al. 2015; Hauzy et al. 2010; Orrock et al. 2013; Vucic‐Pestic, Birkhofer, et al. 2010; Vucic‐Pestic, Rall, et al. 2010). Studies have demonstrated that increasing habitat complexity can lower predation pressure on prey, thus reducing the top‐down control exerted by predators (Chang and Todd 2023; Kalinkat et al. 2013; Mocq et al. 2021). Yet, habitat complexity could also aid predators in their search for prey, particularly those operating in three‐dimensional environments (i.e., species that search a habitat volume rather than a surface for prey (Pawar et al. 2012)), as observed in the case of a freshwater copepod preying on ciliates (Reiss and Schmid‐Araya 2011). Therefore, habitat complexity can potentially modify or shape energy flows through food webs, influencing feeding relationships between resources and consumers and among predators and prey.

Functional response models are a suitable tool when it comes to addressing the effects of habitat complexity on species interactions, as they describe the relationship between prey density and the intake of prey by a predator (Holling 1959a; Li et al. 2018). More generally, functional response models have been central in understanding interaction strengths, population dynamics, and ecosystem stability (Berlow et al. 2009; Kratina et al. 2022; Rall et al. 2008; Williams and Martinez 2004).

Characterizing an organism's feeding behavior in short‐term experiments typically involves examining how feeding rate varies with prey density. The feeding rate is determined by the time a predator requires to locate, attack, capture, handle, ingest, and digest prey (Holling 1959a; Li et al. 2018). When measured across a gradient of increasing prey density, feeding rates often follow a hyperbolic saturating curve, described as a type II functional response. The initial rise in feeding rate is governed by the attack rate, originally termed the “instantaneous rate of discovery” by Holling (1959a), and is also referred to in the literature as capture rate, filtration rate, or search rate. The asymptotic portion of the curve is constrained by handling time, which reflects the period required to capture, subdue, and consume a prey item, during which the predator is unable to forage for additional prey (Li et al. 2018). Beyond the type II response, alternative patterns of functional responses are observed. For example, Holling (1959b) described an s‐shaped curve, or type III functional response, in mammalian predators under field conditions. In this case, both feeding rate and attack rate are functions of prey density, with predators exhibiting improved efficiency as prey become more abundant. Undeniably, the experimental design is critical in identifying functional response patterns accurately (DeLong et al. 2025; Sarnelle and Wilson 2008), and habitat presence might be a particularly influential predictor in functional response experiments (Kalinkat and Rall 2015). For example, habitat complexity has frequently been cited as a key driver of type III functional responses (Barrios‐O'Neill et al. 2015; Kalinkat et al. 2013; Vucic‐Pestic, Birkhofer, et al. 2010; Vucic‐Pestic, Rall, et al. 2010).

Testing how refuge availability and habitat complexity alter trophic relationships requires careful experimental design to avoid confounding structural quantity with habitat complexity (Flores et al. 2016; Pierre and Kovalenko 2014). For instance, in a laboratory experiment with a centipede predator and springtail prey, Kalinkat et al. (2013) reported that increasing structural complexity through the addition of leaves increased surface area for both predator and prey but did not provide effective refuge. The associated changes in space‐dependent functional response parameters were independent of surface area, indicating a dilution effect rather than refuge use (Kalinkat et al. 2013). Importantly, habitat complexity can increase not only by adding more structures, but also through changes in the spatial arrangement of a fixed number of elements (Flores et al. 2016). Furthermore, certain forms of habitat complexity can represent obstacles without contributing to available surface area (Hauzy et al. 2010).

In addition to habitat complexity, the predation strategy of the predator is an important factor determining the interaction strength of predator and prey (Almany 2004; Schmitz 2017) and, by extension, the stability of food webs. These strategies include ‘ambush’ (also known as ‘sit‐and‐wait’ tactics) and ‘pursuit’ (active chasing), and these traits can determine spatial utilization and interactions with environmental structures (Pawar et al. 2012; Schmitz 2017). For instance, ambush predators, exemplified in aquatic systems by species like dragonfly and damselfly nymphs, occur in complex habitats that provide ample hiding spots, enhancing their ability to remain concealed and ambush prey (Chang and Todd 2023; Mocq et al. 2021; Soukup et al. 2022). An example of the latter is *Ischnura elegans*, a damselfly species that is abundant and common across Europe. It inhabits both running and standing waters and can tolerate brackish and polluted water (Mikolajewski et al. 2015). The larvae are known to exhibit functional response to *Daphnia magna* as prey and to have a broad diet spectrum, including isopods (Thompson 1978a, 1978b).

Conversely, pursuit predators, such as predatory fish, certain types of predatory shrimp, or backswimmers, require more open and less obstructed spaces to effectively chase down their prey. Dense structures within complex habitats, however, can impede their navigation and reduce hunting efficiency (Almany 2004; Warfe and Barmuta 2006). This generalization is likely to manifest differently depending on habitat type and species‐specific traits. Within the genus *Notonecta*, for example, species exhibit distinct adaptations to habitat use (Giller and McNeill 1981; Svensson et al. 2000). For instance, *Notonecta glauca* is an actively swimming, suction‐feeding backswimmer species that occupies a wide range of freshwater habitats across Europe (Giller and McNeill 1981). This abundant predator (Svensson et al. 2000) has been hypothesized to preferentially exploit open‐water habitats (Gittelman 1974), yet structurally complex ones containing plants, where individuals perch before pursuing prey (Giller and McNeill 1981).

Therefore, habitat complexity is likely to have varying impacts on predatory success depending on the predator's hunting strategy. For instance, in aquatic environments, a more complex habitat may favor ambush predators by providing increased hiding opportunities, while posing challenges for active swimming predators, thereby potentially reducing their hunting success (Almany 2004; Dunn and Hovel 2020; Froneman and Cuthbert 2022).

Studying how the functional response of different predators is modulated by habitat structure is crucial to understanding trophic interactions and energy transfer through real food webs in nature. Indeed, Pawar et al. (2012) point out that an important future direction in ecological research is to understand how habitat complexity affects search‐ and consumption rates. The rationale is that predicting the strengths of pairwise trophic interactions is fundamental for understanding higher‐order effects, such as indirect interactions and omnivory; and, ultimately, for explaining stability and diversity in ecological networks (Berlow et al. 2009; Pawar et al. 2012). We aimed to explore how two different predators ( *I. elegans* and *N. glauca* ) with contrasting hunting strategies are affected by habitat complexity in their search and consumption of prey. Specifically, we tested how habitat complexity (measured as (1) the presence of structure, (2) the amount of structure, and (3) the spatial arrangement of structure) influenced functional response parameters, addressing three hypotheses as follows.

1. Habitat complexity alters the shape of the functional response curve, because complex habitats reduce the detection of prey compared to less complex environments, particularly at low prey densities.
2. Feeding rates generally decrease with increasing amount of habitat structure, resulting in altered functional response parameters. We expected that attack rate rather than handling time would be affected by habitat complexity, meaning the feeding rate would be reduced at low prey densities.
3. The open‐water predator ( *N. glauca* ) prefers less complex environments and hence shows a different response to habitat structure compared to the ambush predator ( *I. elegans* ).

## 2 Materials and Methods

### 2.1 Experimental Setup

The experiment was carried out using individual microcosms (9.5 cm diameter and 11.5 cm high circular beakers). The prey (water hog‐louse *Asellus aquaticus* ) was provided with leaves to feed on. Hence, air‐dried alder leaves ( *Alnus glutinosa* L.) were weighed, added to each microcosm (2 g each), and conditioned in 500 mL water (1 part of filtered pond water to 5 parts of tap water) for 7 days. The water was renewed when the prey was introduced to the microcosms. It should be noted that the individuals were not able to hide in the leaves, and we, therefore, ignored the leaves in any estimates of habitat complexity. Every microcosm was served by one air‐stone, and it was sealed with cling film to prevent evaporation. The experiment was run in a temperature‐controlled room (15°C) with a 14 h light:10 h dark cycle.

**FIGURE 1:**
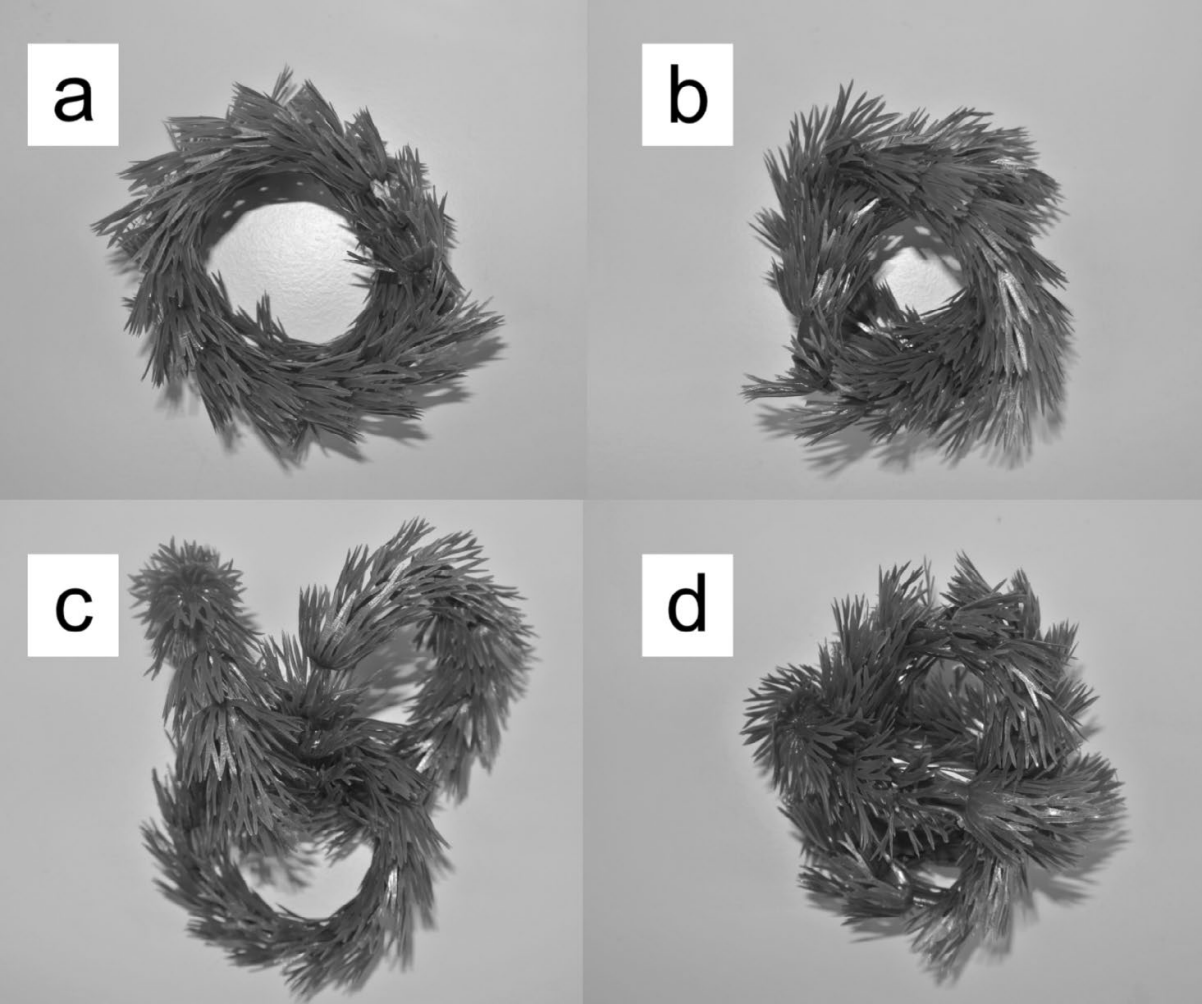
Photographs of the green structures used to create habitat complexity in microcosms with ‘structure present’ (there were also microcosms without any structure added). The basic unit of each structure was a plastic plant strip (mimicking *Ceratophyllum* spp.), joined up as a ring (~8 cm in diameter). Four levels of fractal dimension were created with them: (a) level 1 consisted of two rings aligned, with a fractal dimension (*D*) of 1.77; (b) level 2 consisted of two rings twisted into each other (*D* = 1.80); (c) level 3 consisted of three rings locked together (*D* = 1.81); and (d) level four was a ball made from 3 rings together (*D* = 1.83). Fractal dimension was used to assign a categorical value for ‘complexity’ to each treatment (zero to four). This design therefore also gave two levels of ‘amount of structure’: 3 g for complexity level 1 and 2 and 4.5 g for complexity level 3 and 4. Figure taken from Flores et al. (2016).

When manipulating habitat complexity, it is important to disentangle the effects of complexity per se from the effects of the amount of structure in the microcosm (see Kalinkat et al. 2013 for detailed reasoning). We used plastic rings to create both structure and different levels of complexity (Figure 1). These rings were artificial plastic plants mimicking the aquatic plant *Ceratophyllum* spp. (Code No. FRF 491, Fish are Fun), assembled into a ring approximately 8 cm in diameter. From these rings, four levels of fractal dimension were created. The simplest configuration, with a fractal dimension of 1.77, consisted of two rings placed in alignment (Figure 1a). A slightly higher dimension (1.80) was obtained by twisting two rings together (Figure 1b), and a dimension of 1.81 was produced by interlocking three rings (Figure 1c). The most complex structure, with a dimension of 1.83, was formed by combining three rings into a ball (Figure 1d). Hence, habitat complexity was created in three ways: (1) as the presence versus absence of habitat structure; (2) as the amount of structure (0, 2 or 3 of plastic plant rings); and (3) as two spatial configurations of structures in the case of 2 and 3 rings (Figure 1; and Flores et al. 2016), resulting in five complexity levels. The order of these five levels (from ‘simple’ to ‘complex’ [levels 0, 1, 2, 3 and 4]) was determined by measuring the fractal dimension in the feeding arena (Figure 1; and Flores et al. 2016). The overall rationale for choosing the structures was to simulate natural conditions in which the predators were able to enter the structures to varying degrees, thereby hindering predation.

### 2.2 Adding Organisms to Microcosms

We tested the feeding functional response of the ambush predator *Ischnura elegans* (van der Linden) (16.8 mm average length and 3.5 mm average head width) in its larval stage, and the pursuit predator *Notonecta glauca* (L.) (14.7 mm average length and 4.9 mm average width) preying on *A. aquaticus* (6.6 mm average body length). *Ischnura elegans* detects prey visually, and possibly via chemical cues, before striking prey with a labial mask, ingesting the prey whole (Mikolajewski et al. 2015). *Notonecta glauca* detects prey using visual and vibratory stimuli and captures prey with its raptorial legs and pierces it (Giller and McNeill 1981). Applying body dimension to dry weight regressions from the literature (Baines et al. 2015; Benke et al. 1999; Reiss et al. 2011), we estimated these species to have an average dry weight of 8.6, 10.5, and 2.1 mg per individual for *I. elegans* , *N. glauca* , and *A. aquaticus* , respectively.

The predators were allowed to feed for 24 h as trials and previous studies (Li et al. 2018; Thompson 1978b) indicated that this timeframe can produce meaningful functional response curves. Further, it reflects one full day and night cycle with all relevant physiological behaviors (Li et al. 2018). All treatments were run at once and replicates were run in time blocks (i.e., on consecutive days). These blocks had the advantage that this approach allowed us to add ‘very high’ density treatments after running the first replicate and to further adjust the number of replicates needed after observing feeding during the first time block. Prey densities for *I. elegans* were 1, 3, 5, 10, 30, 80, and 120 individuals per microcosm. Prey densities for *N. glauca* were 1, 3, 5, 10, 30, 80, 120, and 180 individuals per microcosm. Replication varied from 1 to 6 replicates per treatment (mostly 3 for *I. elegans* and 6 for *N. glauca* ), and in total, we ran 297 microcosms. The data are available on GitHub (https://github.com/b‐c‐r/CRITTERdata) and on Zenodo (Flores et al. 2025).

### 2.3 Functional Response Equations

**FIGURE 2:**
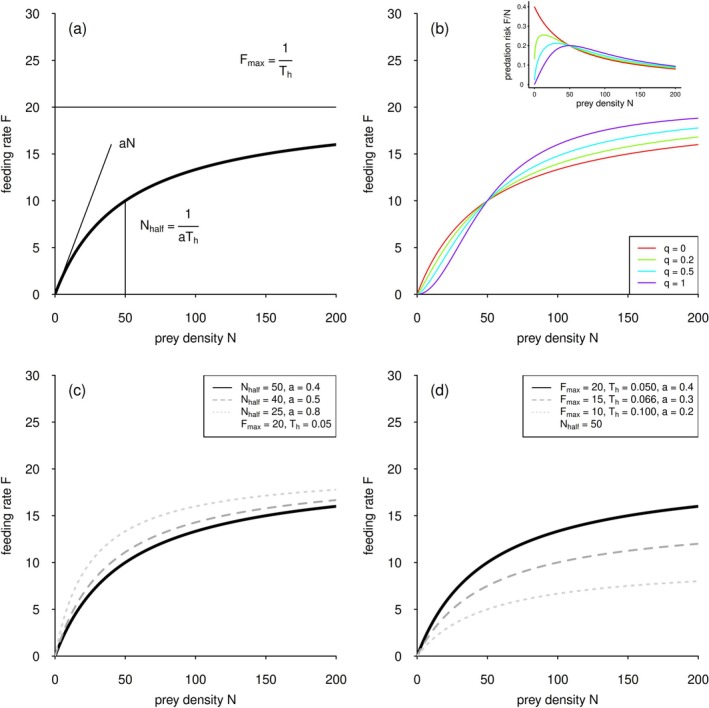
(a) The type II functional response (bold line) is either controlled by the parameters attack rate, *a*, and handling time, *T*_*h*_ (Holling 1959b), or the maximum feeding rate *F*_max_ and the half‐saturation density *N*_half_ (Real 1977). (b) The generalized functional response model allows for a seamless shift between the hyperbolic type II functional response (*q* = 0) and a ‘strict’ type III functional response (*q* = 1). Inlay: The predation risk of a single prey item decreases in the case of a hyperbolic type II functional response (*q* = 0); when the function becomes s‐shaped (type III), the predation risk increases with increasing prey density, leading to small but stable populations. (c) Example where the Holling and Real models (shown in [a]) are interchangeable. The feeding rate decreases primarily at low prey densities. Although it appears that the maximum feeding rate also declines, this is misleading; in reality, the maximum rate is simply reached at much higher prey densities. (d) Example where the models are not interchangeable. The variation in feeding rates as a function of parameter values is clearly visible. The feeding rate decreases uniformly across all prey densities, that is, the decline is proportional and independent of prey density.

Characterizing an organism's feeding behavior (in short‐term experiments), feeding rate, *F*, is a function of prey density, *N*, and determined by the time required for predators to find, attack, capture, handle, ingest, and digest their prey (Holling 1959a; Li et al. 2018). When observing feeding rate for a range of increasing prey density, these rates often have a hyperbolic saturating shape, called the type II functional response (Figure 2a). The initial increase in the feeding rate, *F*, is driven by the attack rate, *a* (Holling 1959a). The handling time, *T*_*h*_, controls the saturation of the curve (Figure 2a; Holling 1959a, 1959b). Alternatively, the function can be written using the maximum feeding rate, $F_{\max}=1/T_{h}$, and the half‐saturation density, $N_{\text{half}}=1/\left(aT_{h}\right)$, that is, the prey density where half of the maximum feeding rate is reached (Real 1977; Figure 2a). Following the ‘Holling approach’ (i.e., models that use attack rate and handling time *sensu* Holling 1959a) and the ‘Real approach’ (i.e., models using maximum feeding rate and half‐saturation density *sensu* Real 1977), the full functional response models are

(1) $$F=\frac{\text{aN}}{1+aT_{h}N}=\frac{F_{\max}N}{N_{\text{half}}+N}$$

In addition to the hyperbolic shape, other functional response patterns are observed, for instance Holling (1959b) reported an s‐shaped functional response for mammal predators under field conditions. The s‐shape (type III response) can be observed when both the feeding and attack rates are a function of prey density, meaning that the predator optimizes hunting prey with increasing prey density. The simplest way to include this feature in the above models is to assume a linear increase in the attack rate, a=bN, with b being the attack coefficient (Juliano 2001). Dubbed the generalized functional response model, this approach allows for a seamless switch from a type II functional response to an s‐shaped type III functional response (Figure 2b), promoted by many modeling studies (Jeschke et al. 2004; Kalinkat and Rall 2015; Rall et al. 2008; Williams and Martinez 2004). Here, attack rate is a power‐law function of prey density ($a=bN^{q}$). If the ‘shape parameter’ q is 0, the functional response is type II, and if the shape parameter q is 1, the functional response is a ‘strict’ type III response (Kalinkat et al. 2023). The generalized functional response models are

(2) $$F=\frac{bN^{1+q}}{1+bT_{h}N^{1+q}}=\frac{F_{\max}N^{1+q}}{N_{\text{half}}^{1+q}+N^{1+q}}$$

The difference between the hyperbolic type II and s‐shaped type III functional response can be explained by the differences in predation risk (Figure 2b). The stabilizing effect of the type III functional response can be due to a low predation risk per prey individual (Figure 2b inlay).

### 2.4 Data Analysis

Equations (1) and (2) assume that prey is replenished; however, in our laboratory experiments, prey density declined through time. The problem of prey depletion (and its possible solutions) is described by Rosenbaum and Rall (2018). Following the latter authors, we used the Rogers Random Predator Equation (Rogers 1972; Royama 1971) modified with the Lambert *W* function (Bolker 2008) to fit type II functional responses, simulating prey decline (Rosenbaum and Rall 2018). For estimating the generalized functional response parameters, we directly fitted simulated time series to our data. These methods prevent biased parameter estimations (see details in Rall, Aranbarri, Flores, de Guzmán, et al. 2025; Rosenbaum and Rall 2018).

We first investigated if the shape of the functional response was hyperbolic or s‐shaped for all 10 treatments (5 complexity treatments and 2 predator species). This was achieved by fitting the generalized functional response model (Equation 2, Figure 2b), allowing for a seamless shift between type II and type III functional responses (Rosenbaum and Rall 2018).

After determining the shape of the functional response, we tested a suite of functional response models to address the effects of habitat on the functional response parameters (see Rall, Aranbarri, Flores, de Guzmán, et al. 2025). This suite of models tested for (1) the assumption that habitat does not influence feeding, (2) an effect of no structure versus structure, (3) an effect caused by the amount of structure, and (4) effects due to the complexity (fractal dimension) of the structure (while being related to ‘amount’). The models targeted two parameters or three parameters (case generalized functional response) at once (Table 1).

**TABLE 1:** Overview of models tested on two parameters at once to find out if the feeding rate was affected by habitat complexity.

	Holling approach									Real approach							
Parameter	*T*_*h*_				*a*					*F*_max_				*N*_half_			
Hypothesis	Zero	One	Two	Three	Zero	One	Two	Three		Zero	One	Two	Three	Zero	One	Two	Three
	Habitat has no effect	Presence has an effect	Amount has an effect	Complexity level has an effect	Habitat has no effect	Presence has an effect	Amount has an effect	Complexity level has an effect		Habitat has no effect	Presence has an effect	Amount has an effect	Complexity level has an effect	Habitat has no effect	Presence has an effect	Amount has an effect	Complexity level has effect
Model									Model								
1H	1	0	0	0	1	0	0	0	1R	1	0	0	0	1	0	0	0
2H	1	0	0	0	0	1	0	0	2R	1	0	0	0	0	1	0	0
3H	1	0	0	0	0	0	1	0	3R	1	0	0	0	0	0	1	0
4H	1	0	0	0	0	0	0	1	4R	1	0	0	0	0	0	0	1
5H	0	1	0	0	1	0	0	0	5R	0	1	0	0	1	0	0	0
6H	0	1	0	0	0	1	0	0	6R	0	1	0	0	0	1	0	0
7H	0	1	0	0	0	0	1	0	7R	0	1	0	0	0	0	1	0
8H	0	1	0	0	0	0	0	1	8R	0	1	0	0	0	0	0	1
9H	0	0	1	0	1	0	0	0	9R	0	0	1	0	1	0	0	0
10H	0	0	1	0	0	1	0	0	10R	0	0	1	0	0	1	0	0
11H	0	0	1	0	0	0	1	0	11R	0	0	1	0	0	0	1	0
12H	0	0	1	0	0	0	0	1	12R	0	0	1	0	0	0	0	1
13H	0	0	0	1	1	0	0	0	13R	0	0	0	1	1	0	0	0
14H	0	0	0	1	0	1	0	0	14R	0	0	0	1	0	1	0	0
15H	0	0	0	1	0	0	1	0	15R	0	0	0	1	0	0	1	0
16H	0	0	0	1	0	0	0	1	16R	0	0	0	1	0	0	0	1

*Note:* In total, four parameters are tested, yet only two of them in one pass, as this reduced the testing from a possible 128 combinations to 32 models. Furthermore, the four parameters correspond to two different simulation models (dubbed ‘H models’ and ‘R models’; after Holling 1959a, Holling 1959b and Real 1977, respectively). For each parameter, there are four hypotheses: ‘Zero’ assumes that habitat has no effect; ‘One’ assumes that presence has an effect (2 levels: present and absent); ‘Two’ assumes that amount has an effect (3 levels: 0, 2, and 3 plastic plant rings) and ‘Three’ assumes that complexity level (fractal dimension) has an effect (5 levels: 0 to 4 complexity levels). 1 = model tests for this; 0 = model does not test for this.

Testing four assumptions on two parameters in a fully factorial fashion gives a suite of 16 models for the ‘Holling’ approach (1H to 16H) and the ‘Real’ approach (1R to 16R) of functional responses each (Table 1). We dubbed the approach using *a* and *T*_*h*_ ‘Holling’ and the approach using *F*_max_ and *N*_half_ ‘Real’ as these authors were the first to introduce these parameters (Holling 1959a, 1959b; Real 1977). Consequently, models 1H to 16H targeted parameters *T*_*h*_ and *a* (*sensu* Holling 1959a, 1959b) and 1R to 16R targeted *F*_max_ and *N*_half_ (*sensu* Real 1977). In case we detected any type III functional response, the full‐factorial scheme would have needed 64 models for each functional response formulation (see Rall, Aranbarri, Flores, de Guzmán, et al. 2025).

In brief, the reasoning for fitting both Holling and Real approaches to functional response models lies in testing for effects of a predictor like habitat complexity. Although Holling and Real equations can be generally translated into each other (Figure 2a, Equations 1 and 2), this does not necessarily work when the parameters are connected to an overarching model, like in our case, habitat complexity levels. For instance, as long as both parameters depend on the same habitat complexity measure (e.g., five levels of habitat complexity), the parameter values of the Holling approach and the Real approach can be calculated from each other. Yet, in some cases, models are not exchangeable, for example, if the attack rate/half‐saturation densities depend on the amount of structural elements, and handling time/maximum feeding rate are fitted to the level of complexity. This phenomenon is caused by the fact that the half‐saturation density is not simply the inverse of the attack rate but also depends on the handling time. A change in the maximum feeding rate, for instance, automatically leads to a proportional change in attack rate if the half‐saturation density is constant (see Figure 2c,d and Rall, Aranbarri, Flores, de Guzmán, et al. 2025 for more details).

This approach enabled testing for these different habitat predictors on all functional response parameters in one pass and avoided confounding ‘amount of structure’ with ‘complexity’, on the basis of the rationale developed in Flores et al. (2016). We compared the models by using both Akaike's Information Criterion (AIC) and the Bayesian Information Criterion (BIC), where the lowest AIC and BIC score indicates the best (most parsimonious) model. The output includes a point estimate for the parameter and lower and upper confidence intervals (CIs).

Hence, the functional response models provide information about the overall amount of prey eaten, driven by the maximum feeding rate or its inverse, the handling time. This effect is most obvious at high prey densities, where the curve reaches its asymptote (Figure 2a,c). The attack rate and the half‐saturation density predominantly control the feeding rate at lower densities (Figure 2a,d), and we were able to compare those parameters between habitat treatments.

We provide the underlying code on GitHub (https://github.com/b‐c‐r/CRITTERcode) and as a citable version on Zenodo (Rall, Aranbarri, Flores, de Guzman, et al. 2025). Further, a statistics report is available (https://github.com/b‐c‐r/CRITTERstatistics; Rall, Aranbarri, Flores, de Guzmán, et al. 2025).

## 3 Results

**FIGURE 3:**
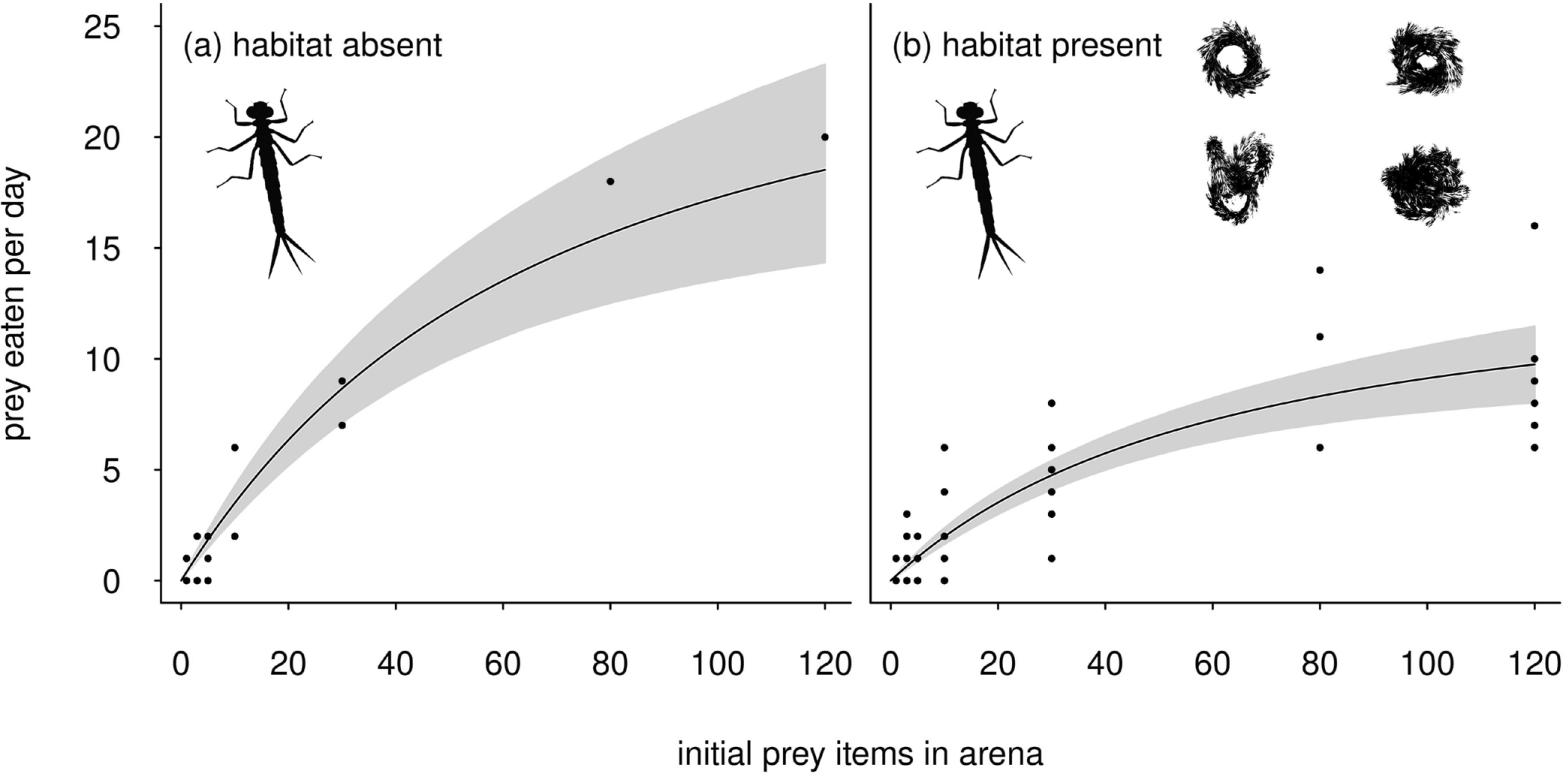
Functional response curves for the ambush predator *I. elegans* feeding in microcosms (arena) without structure (a) and in arenas with plastic plant structures (b). The right panel (b) shows data across four habitat complexity levels because the most significant statistical model for the *I. elegans* feeding pattern was the difference between complexity level zero (no structure) and treatments with structure present. Gray shaded areas denote the 95% confidence intervals.

**FIGURE 4:**
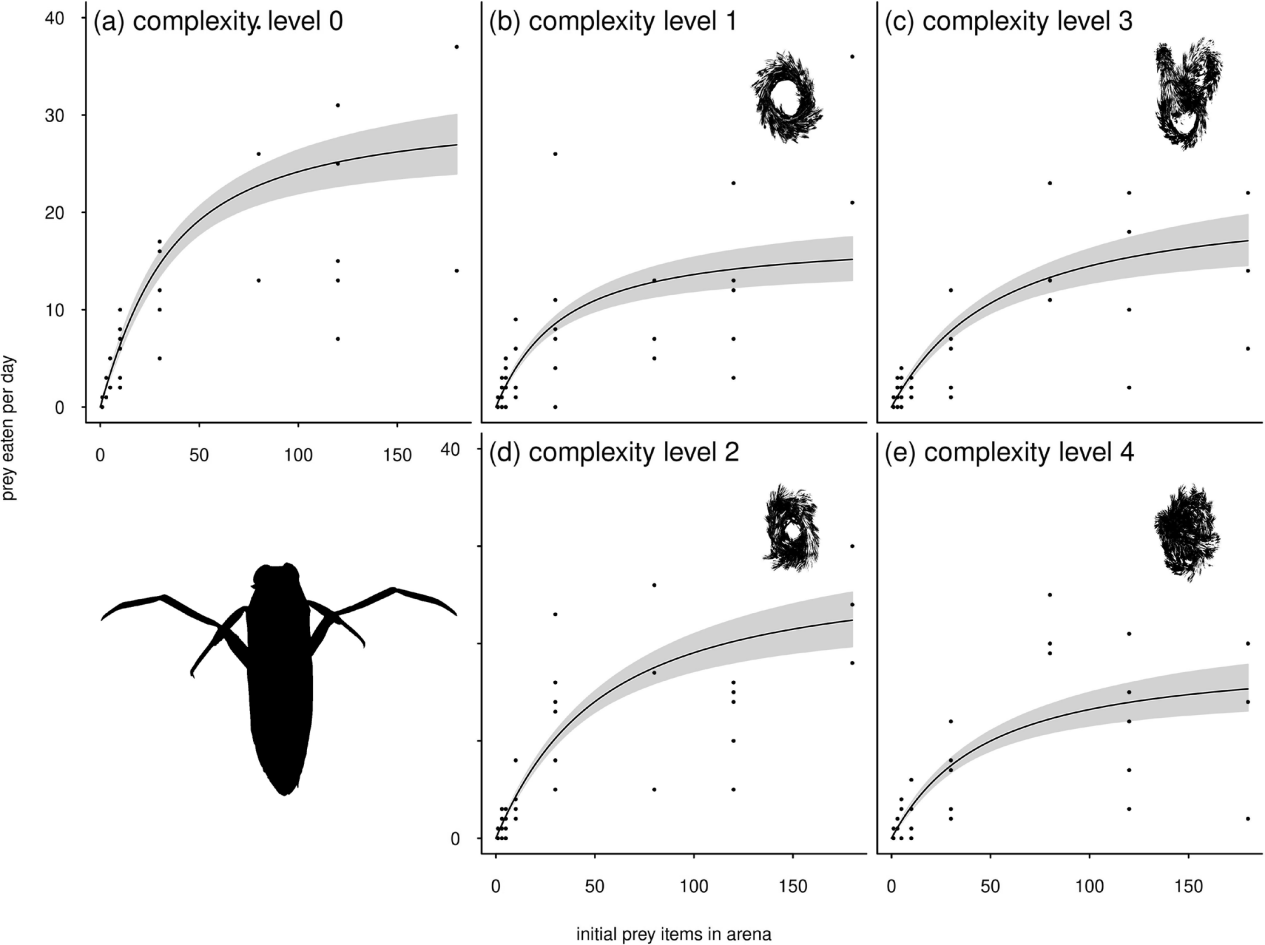
Functional response curves for the pursuit predator *N. glauca* in feeding arenas with different complexity levels created with plastic plant rings (see images in panels b–e). Gray shaded areas denote the 95% confidence intervals.

The feeding rate of both predators initially increased with prey density, but then leveled off at a plateau (Figures 3 and 4). The shape of the functional response curves for both *N. glauca* and *I. elegans* (Figures 3 and 4) consistently fit best to a type II model for all five complexity levels, as established by fitting the generalized functional response model to the data (Table 2). Applying this model to both species and all complexity levels, the shape parameter *q* was never significantly different from zero; hence, feeding rates followed a functional response type II (Table 2).

**TABLE 2:** Testing for the functional response type for two predators feeding at five complexity levels (0–4), by using the generalized functional response model.

Predator	Complexity	*q*	Significance	Type
*Ischnura elegans*	0	0.116	n.s.	II
*Ischnura elegans*	1	0.047	n.s.	II
*Ischnura elegans*	2	−0.001	n.s.	II
*Ischnura elegans*	3	−0.001	n.s.	II
*Ischnura elegans*	4	0.193	n.s.	II
*Notonecta glauca*	0	−0.011	n.s.	II
*Notonecta glauca*	1	−0.006	n.s.	II
*Notonecta glauca*	2	0.194	n.s.	II
*Notonecta glauca*	3	−0.002	n.s.	II
*Notonecta glauca*	4	−0.001	n.s.	II

*Note:* The proportion of prey eaten declined with increasing complexity level. The shape parameter *q* was not significantly different from zero in all cases; hence, the feeding rate followed a functional response type II.

Therefore, we tested a total of 32 type II functional response models to test for the effect of habitat. The overall feeding rate of *I. elegans* was mainly explained by the absence versus presence of structure, evidenced by the functional response exhibited by this species (Table 3, Figure 3). For *I. elegans* , the best fitting model (both lowest AIC and BIC, Table 3) was ‘model 5R’ (Table 3, Figure 3). This model assumed that the effect of structure presence versus absence overrides all other predictors. In the ‘5R’ model, *F*_max_ takes a value of 28 prey eaten in 24 h when no structure is present (CI: 19 to 41). However, when structure was present, *F*_max_ was 15 (CI: 11 to 20). *N*_half_ was not affected by the habitat and took the value of 57 prey items per arena (CI: 34 to 95).

**TABLE 3:** The six best models (see Table 1 for explanation of each model) that describe the functional response parameters *T*_*h*_ and *a* (model names include an ‘H’), or *F*_max_ and *N*_half_ (model names include an ‘R’) for predators *I. elegance* and *N. glauca* feeding on *A. aquaticus* .

AIC			BIC		
Model	df	dAIC	Model	df	dBIC
*Ischnura elegance*					
**Model 5R**	**3**	**0.000**	**Model 5R**	**3**	**0.000**
Model 7R	4	0.265	Model 5H	3	1.583
Model 6H	4	0.952	Model 7R	4	2.731
Model 6R	4	0.952	Model 6H	4	3.418
Model 5H	3	1.583	Model 6R	4	3.418
Model 13R	6	2.107	Model 7H	4	4.718
*Notonecta glauca*					
**Model 15H**	**7**	**0.000**	**Model 15H**	**7**	**0.000**
Model 16H	10	1.274	Model 9R	3	1.004
Model 16R	10	1.274	Model 11H	4	1.485
Model 15R	7	1.990	Model 11R	4	1.485
Model 14R	7	4.189	Model 13R	6	1.507
Model 13R	6	4.854	Model 15R	7	1.990
Model 12H	7	8.352	Model 7H	4	2.516

*Note:* The full suite of models can be found in Rall, Aranbarri, Flores, de Guzmán, et al. (2025). The best fit of the models was tested using AIC (AIC generally performs better for models that have more parameters) and BIC. A low value indicates the best fit. The most significant model is highlighted in bold.

In the case of *N. glauca* , the feeding rate was strongly dependent on habitat complexity, with the predator showing a unique maximum feeding rate (i.e., the inverse of the handling time) for each complexity level and a decreasing attack rate with increasing amount of habitat (Table 3 , Figure 4). The best fitting (most parsimonious) model (both lowest AIC and BIC) for *N. glauca* was ‘model 15H’ (Table 3, Figure 4), and this model assumed that each complexity level has a unique handling time, *T*_*h*_, and that attack rate, *a*, decreases with increasing amount of habitat structure. Hence, the parameters that influence the feeding rate are more complex in this case. The maximum feeding rate (estimated from the handling time (Fmax=1Th)) was 31 (CI: 26 to 36) prey eaten in 24 h for complexity level 0 (no structure), 18 (CI: 15 to 21) for level 1, 28 (CI: 24 to 33) for level 2, 22 (CI: 18 to 27) for level 3, and 19 (CI: 15 to 23) for level 4 (Figure 4). The attack rate of *N. glauca* (Figure 4) decreased with increasing number of habitat structural elements (rings, $\log_{10}\left(a\right)=a_{\text{intercept}}+a_{\text{slope}}N_{\text{rings}}$), indicated by a negative slope (−0.144, CI: −0.191 to −0.097).

**FIGURE 5:**
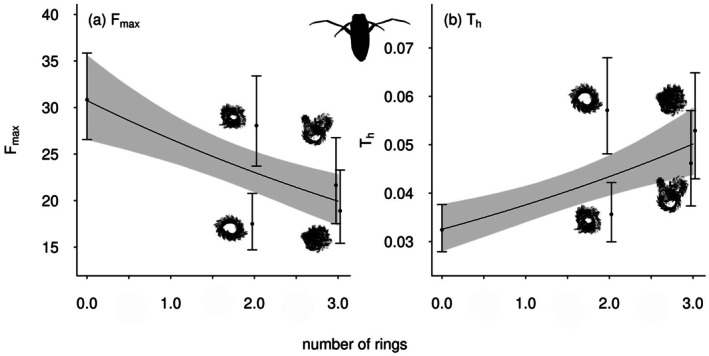
The functional feeding response parameters (a) *F*_max_ and (b) *T*_*h*_ of *N. glauca* feeding on *A. aquaticus* for model 15H (points with whiskers) and the next less complex comparable model 11H (lines and gray areas). The amount of structure (number of plastic plant rings) is regressed against maximum feeding rate (a) and handling time (b). Gray shaded areas denote the 95% confidence intervals.

This more complex model was selected for *N. glauca* over the very similar, but simpler, ‘presence of structure’ model, because ‘unexpected’ responses were observed when two plastic plant rings had been added to the microcosms, both in terms of feeding‐ and attack rate (Figure 5). Evidently, the configuration of two rings produced two distinct structural arrangements, each generating different habitat types. In contrast, the configuration with three rings consistently resulted in a compact structure resembling a dense ball, which functioned as a uniformly suitable hiding space. In the case of two rings, one configuration (complexity level 2, two twisted rings; see Figure 2, panel 2) provided more open space, allowing the pursuit predator *N. glauca* greater room to navigate (Figure 5).

Overall, fitting these models showed that prey consumption was considerably less for both predators when complex structures were present, compared to the no habitat structure environment (Figures 3 and 4). For instance, the maximum number of prey consumed was less than half in some ‘no structure’ versus the ‘structure’ treatments (Figures 3 and 4). To illustrate this difference, in ‘no structure microcosms’, and at a prey density of 120 individuals, the maximum number of prey consumed was 20 and 49 for *I. elegans* and *N. glauca*, respectively. In contrast, at the same prey density, *I. elegans* and *N. glauca*, respectively, fed on a maximum of 9 and 20 *A. aquaticus* in at least one of the structure treatments (the latter were different treatments for the two species; see Figures 3 and 4).

## 4 Discussion

Our findings suggest that habitat complexity affects predator–prey interactions in freshwater ecosystems. Results from our feeding experiments showed that the feeding functional response type did not significantly vary with habitat complexity. While the form of the functional response (type II) remained constant, the magnitude of feeding (*F*_max_, attack rate, and handling time) decreased in microcosms with structures. We found support for the hypothesis that increased habitat complexity would decrease feeding rates overall. Both the ambush predator (damselfly larvae *Ischnura elegans*) and the pursuit predator (backswimmer *Notonecta glauca* ) showed higher feeding rates in microcosms without structures compared to those with structure. To illustrate, at a prey density of 120 prey items, average consumption reached over four ( *I. elegans* ) and eight ( *N. glauca* ) times the predator body weight in 24 h (20 and 33 prey eaten on average by *I. elegans* and *N. glauca*, respectively). The latter assumes that prey was ingested whole (which was not the case for *N. glauca* , which left prey body parts after feeding). Taken together, this suggests that complexity affected energy flow considerably and that, although habitat complexity reduced feeding rates by affecting *F*_max_, attack rate, and handling time, the overall mechanisms (shape of the functional response curve) remained consistent across different complexity levels.

Generally, it is known that complex structures offer refuge for prey, thereby reducing the efficiency of predators (Froneman and Cuthbert 2022; Hauzy et al. 2010; Mocq et al. 2021). This reduction in feeding rates due to increased habitat complexity implies a decrease in the top‐down control of predators on prey populations (Chang and Todd 2023). In our study, the reduction in feeding rates was associated with both a reduction in the attack rate and an increase in handling time (i.e., an increase in maximum feeding rate while the half‐saturation density was relatively constant). However, the magnitude of these changes varied across different complexity levels. Previous studies, conducted using microcosm approaches, have shown that attack rate and handling time can respond differently to habitat complexity. For instance, a study on the zooplankton prey *Paracartia longipatella* and the predator *Mesopodopsis wooldridgei* reports strong complexity effects on attack rates (Froneman and Cuthbert 2022 ). Yet, the dragonfly larva *Aeshna cyanea* preying on *Chaoborus obscuripes* larvae was shown to change handling time with complexity (Mocq et al. 2021). Soil mites preying on collembolans displayed varying responses in both attack rate and handling time with habitat structure (Hauzy et al. 2010).

Overall, these previous studies also demonstrate that whether habitat is perceived as ‘complex’ by the organisms is context‐dependent and that the size proportions of predator to prey—and to habitat—are a key factor. For instance, in feeding experiments with the predatory nematode *Prionchulus muscorum*, the addition of structure had little effect as predator and prey had the same body shape and mobility—hence the feeding pattern was mainly driven by prey size (Kreuzinger‐Janik et al. 2019). Estimating fractal dimension to assign an ‘objective’ complexity level (i.e., a score from low to high) proved valuable for our experiment (see also Flores et al. 2016), but it was obvious that one of the predators ( *N. glauca* ) was more successful than expected in one case because the structure had much open space, despite its high fractal dimension. Therefore, finding a metric for habitat complexity should include spatial arrangement and consider the dimensions of prey, predator, and habitat structure.

Clearly, it is important to add this form of realism in laboratory experiments as abiotic factors, such as habitat structure (e.g., Flores et al. 2016) or temperature (e.g., Sentis et al. 2022), can change the outcomes of feeding experiments. A more complex physical structure could have a positive effect on the overall performance of an assemblage (Flores et al. 2016) because species can feed in their optimum environment (in analogy to their optimum temperature) while predation is dampened. The amount or arrangement of physical structure could, therefore, influence consumer effects and could be an important predictor to consider in ecological research that focuses on energy transfer and ecosystem functioning (Flores et al. 2016).

We hypothesized that at the lowest habitat complexity (i.e., microcosms without added plastic plant structures), the shape of the functional response would best fit a type II functional response because feeding rates would be mainly limited by the time required by the predator to kill and eat its prey. With increasing habitat complexity levels, we expected a sigmoid functional response (i.e., type III functional response), as at low prey densities the efficiency of the predator would be limited by the structures offering multiple hiding places (Kalinkat et al. 2013; Vucic‐Pestic, Birkhofer, et al. 2010; Vucic‐Pestic, Rall, et al. 2010). However, as prey density increases and the amount of refuge becomes saturated, the efficiency of the predator would again increase at intermediate prey densities (Figure 2b,c).

Yet, the functional response of both predators was consistently best described by a type II model across all the levels of habitat complexity. Mikolajewski et al. (2015) highlighted a phenotypic plasticity for *Ischnura*, allowing it to adapt to environmental changes while maintaining a type II functional response. Moreover, our design of the habitat structure allowed both predators to enter the potential prey refuge, which may also be a reason for not finding a type III response. One prey per predator was the lowest density in our experiment, which corresponds to a density of 140 individuals per square metre, but in nature, lower abundances can be observed for many taxa (Larrañaga et al. 2009). Indeed, not considering low prey in laboratory set‐ups can hinder the detection of a type III response (Sarnelle and Wilson 2008). Although the prevalence of the type III functional response has been postulated as the way to maintain stability of prey populations and high diversity (Kalinkat et al. 2023; Murdoch and Oaten 1975; Rall et al. 2008), type II is still the most common functional response in feeding studies (DeLong et al. 2025; Dunn and Hovel 2020; Kalinkat and Rall 2015). Even though a type III functional response was not supported by our data, we saw a hint that the interaction of both predators and their prey was stabilizing, as feeding rates generally decreased with the presence and with increasing complexity of the habitat structure. The general decrease in interaction strength is associated with more stable population dynamics and higher species richness in model food webs (May 1972; Rall et al. 2008; Yodzis and Innes 1992).

The two predators in our experiment differed in terms of their predatory strategies, and the functional response parameters highlighted some of those differences. The pursuit predator (the backswimmer *N. glauca* ) exhibited three times the attack rate of the ambush predator (the damselfly *I. elegans* ), although a similar handling time, when no structures were present in their environment. Furthermore, as an open‐water hunter, *N. glauca* is more likely to operate in a 3D environment (Pawar et al. 2012) compared to *I. elegans* , which sits within or on the habitat surface. Indeed, the dimensionality of consumer search space is probably a major driver of species coexistence and the stability and abundance of populations (Pawar et al. 2012).

As predicted, the effect of habitat complexity was stronger for the pursuit predator than the ambush predator. An explanation is the barrier effect that the structures can create for the pursuit predator, reducing the visibility of prey and the capture success (Grabowski and Kimbro 2005). In agreement with Svensson et al. (2000), habitat heterogeneity significantly reduced predation efficiency in *N. glauca* , highlighting the vulnerability of pursuit predators to habitat complexity. Previous studies have also shown that *Notonecta* spp. are more successful in open environments (Gittelman 1974), and that factors such as water depth and refuge availability influence their hunting behavior (Cockrell 1984).

Our short‐term experiment with three species certainly demonstrated that habitat complexity is a key factor to be considered when it comes to trophic interactions. Both predators were more successful in environments without structure and were able to feed on substantially more prey in this open environment. We conclude that habitat complexity has the potential to shape species interactions and carbon flow within the food web. The results of this study demonstrate that increased habitat complexity significantly reduces feeding rates in both *I. elegans* and *N. glauca* . The reduction in predation efficiency, driven by decreases in attack rates and increases in handling times, supports the idea that structural complexity provides refuge for prey, limiting predator success. Despite these changes in feeding rates, both species maintained a type II functional response across all levels of habitat complexity, indicating that although habitat structure affects predator efficiency, it does not fundamentally alter the shape of the functional response curve. Overall, these findings underscore the critical role of habitat complexity in shaping trophic interactions and sustaining energy flow. In more complex, natural ecosystems, habitat complexity will drive species coexistence, stability of food webs, and diversity of communities.

## Author Contributions

**Mireia Aranbarri:** formal analysis (supporting), visualization (supporting), writing – review and editing (equal). **Lorea Flores:** conceptualization (supporting), investigation (lead), writing – original draft (equal). **Ioar de Guzmán:** data curation (supporting), formal analysis (supporting), visualization (supporting), writing – review and editing (supporting). **Aitor Larrañaga:** conceptualization (supporting), data curation (equal), supervision (supporting), writing – original draft (equal), writing – review and editing (equal). **Björn C. Rall:** data curation (equal), formal analysis (lead), visualization (lead), writing – review and editing (equal). **Julia Reiss:** conceptualization (lead), data curation (equal), formal analysis (supporting), investigation (supporting), methodology (lead), supervision (lead), writing – original draft (supporting), writing – review and editing (lead).

## Conflicts of Interest

The authors declare no conflicts of interest.

## Data Availability

The datasets analyzed for this study can be found on GitHub (https://github.com/b‐c‐r/CRITTERdata), where we also provide the code (https://github.com/b‐c‐r/CRITTERcode) and statistical methods (https://github.com/b‐c‐r/CRITTERstatistics) for the analysis. Citable versions can be found on Zenodo (https://doi.org/10.5281/zenodo.15348769, https://doi.org/10.5281/zenodo.15346225 and https://doi.org/10.5281/zenodo.15348995).
